# Nanoscale ultrafast dynamics in Bi_2_Te_3_ thin film by terahertz scanning near-field nanoscopy

**DOI:** 10.1016/j.isci.2025.111840

**Published:** 2025-01-20

**Authors:** Ziyu Huang, Jing Li, Peiyan Li, Lin Du, Mingcong Dai, Jiahua Cai, Zejun Ren, Tianxiao Nie, Xiaojun Wu

**Affiliations:** 1 Hangzhou International Innovation Institute, Beihang University, Hangzhou 311115, China; 2 School of Electronic and Information Engineering, Beihang University, Beijing 100191, China; 3 Fert Beijing Institute, MIIT Key Laboratory of Spintronics, School of Integrated Circuit Science and Engineering, Beihang University, Beijing 100191, China; 4 Zhangjiang Laboratory, Shanghai Advanced Research Institute, Chinese Academy of Sciences, Shanghai 201204, China; 5 Wuhan National Laboratory for Optoelectronics, Huazhong University of Science and Technology, Wuhan 430074, China

**Keywords:** Physical chemistry, Quantum physics, Quantum theory

## Abstract

Ultrafast laser interactions with topological insulators (TIs) have garnered tremendous interest for understanding light-matter interactions and developing optoelectronic devices across visible to terahertz (THz) regions owning to their high carrier mobility and sensitivity to electric fields. In particular, within the THz regime, TIs hold considerable promise to realize advanced emitters, modulators, and detectors because of their fascinating ultrafast THz dynamics. However, a detailed understanding of TIs’ THz dynamics necessitates access to both nanoscale and femtosecond timescale. By utilizing THz scattering-type scanning near-field optical microscopy, we investigated THz time-domain spectroscopy, optical-pump THz probe, and THz emission in TI thin films at nanoscale. We analyzed their static scattering characteristics, morphology, and observed THz emission and photocarrier dynamics lasting approximately 10 ps, with dependencies of thickness and power. Our findings reveal the nanoscale ultrafast dynamics of TIs and demonstrate THz s-SNOM’s potential to enhance compact THz device development.

## Highlights

• We revealed morphology of Bi_2_Te_3_ with the nanoscale spatial resolution
• We push the optical-pump THz-probe into the nanoscale of Bi_2_Te_3_
• We observed nanoscale ultrafast THz emission from Bi_2_Te_3_

## Graphical abstract

**Figure:**
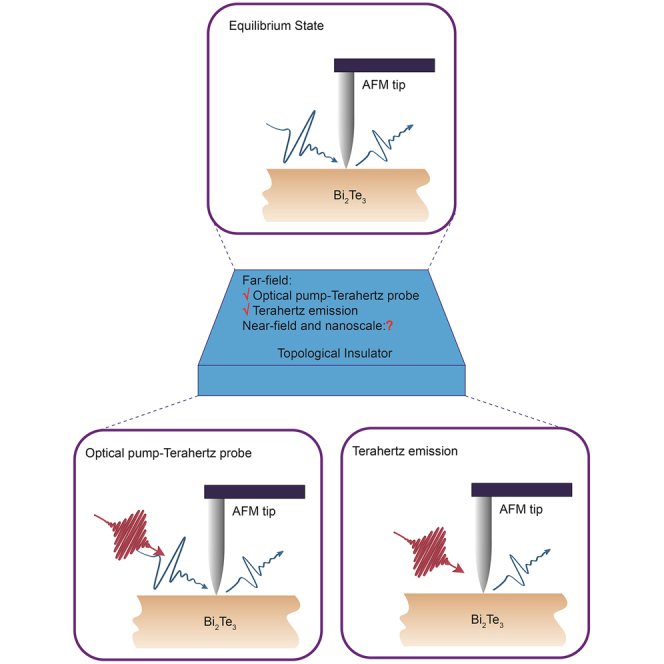

## Introduction

Three-dimensional (3D) topological insulators (TIs) have attracted a lot of interest and enhanced our understanding in the new quantum state of material. Originated from their peculiar electronic structures,^1^ TIs hold the properties of suppressing backscattering,^2^ sensitivity to the electric field,^3^ extremely high carrier mobility,^4^ and robustness against static disorder.^5^ These properties make TIs an ideal platform for the next generation of quantum computing,^6^ spintronic and optoelectronic devices.^7^^,^^8^ Especially in terahertz (THz) regime,^9^^,^^10^^,^^11^ TIs can be designed to achieve the rectification of THz radiation,^8^^,^^12^^,^^13^^,^^14^ function as THz emitter,^9^ and realize strong high-harmonic generation.^15^^,^^16^ However, to facilitate the development of more efficient THz devices, it is crucial to understand their ultrafast phenomena and mechanisms in both macroscopic and microscopic scale. In macroscale, TIs display a wealth of intriguing ultrafast dynamics, such as optically excited coherent phonons,^17^ ultrafast surface currents,^18^ phonon-assisted carrier scattering,^19^ chiral THz generation,^20^ and THz plasmon excitation.^21^ On the contrary, although the nanoscopic ultrafast THz dynamics of TIs is a rich realm as well, it has been largely left unexplored due to the diffraction limit.

In recent years, a strong and advanced technology has been developed to understand ultrafast dynamics in nanoscale, which is ultrafast THz scattering-type scanning near-field optical microscope (ultrafast THz s-SNOM). Ultrafast THz s-SNOM is designed on the basis of the THz s-SNOM, which proves highly useful for the research on plasmons,^22^ polaritons,^23^ and hyperspectral imaging of biomaterials^24^ in THz region with the ability to exceeds the diffraction limit.^25^ Ultrafast THz s-SNOM not only has the functions of static THz s-SNOM, namely THz time domain spectroscopy (THz-TDS), but also has the time-resolved spectral analysis function, that is, the functions of optical pump-terahertz probe (OPTP) and THz emission spectroscopy (TES). The significant leap from THz s-SNOM to ultrafast THz s-SNOM has brought forth highly valuable ultrafast information in THz regime at nanoscale, including carrier dynamics in InAs nanowires,^26^ exciton phase transition mechanism in 2D transition metal dichalcogenides (TMDs),^27^ ultrafast spin currents with nanoscale spatial resolution^28^ and nonlocal diffusion of photoexcited charge carriers in semiconductor.^29^ However, thus far, relevant reports of TIs have primarily focused on characterizing the local scattering properties^30^^,^^31^ and polariton imaging,^32^ with the ultrafast THz dynamics of TIs at nanometer resolution is rarely reported.

In this work, we utilized nanoscopic THz-TDS to study the static scattering feature and morphology of TI, observed THz emission and photocarrier dynamics by TES and OPTP. These ultrafast, sensitive, and contactless measurements provide enhanced dimensional complementarity on TI with a lateral spatial resolution of ∼60 nm.^28^ Through THz-TDS, we captured its optical characteristic of TI materials in the 0.4–1.3 THz range, providing rich spectral data for understanding its electronic structure. OPTP measurement revealed the transient carrier dynamics of TI under laser excitation, visually taking the snapshots of the maximum scattered THz signals with different pumping delay in spatial distribution to manifest the carrier relaxation process which can be regarded as a strong *in situ* characterization method, and TES has further advanced our understanding of the ultrafast physical mechanism of TI. Our research results indicate that the ultrafast THz s-SNOM technology is a unique and powerful means to explore the local ultrafast dynamic information of nanomaterials such as TI with high resolution and sensitivity. We believe that ultrafast THz s-SNOM can enable us to discover phenomena in advanced nanomaterials and accelerate the development of THz nano-devices.^33^

### Experimental and sample

#### Experimental setup

**Figure 1:**
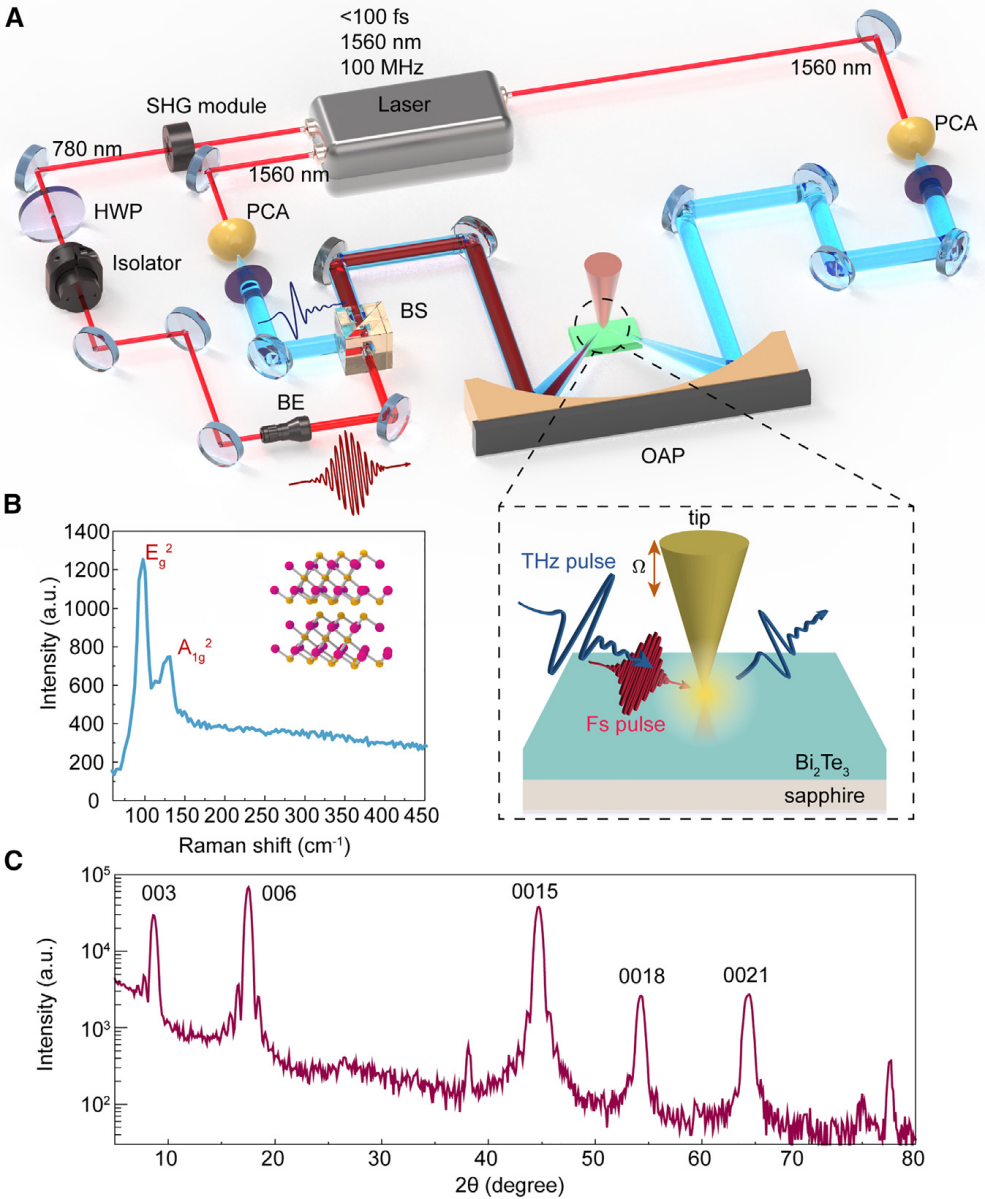
Schematic diagram of s-SNOM and sample characterization (A) The experimental setup of s-SNOM, HWP: half-wave plate, SHG module: second-harmonic generation module, BE: beam expander, BS: beam splitter, PCA: photoconductive antenna, OAP: off-axis parabolic mirror. The blue beams stand for THz propagation route. The red beams stand for laser propagation route. On the left side, the PCA is used as THz emitter to generate probe signal. On the right side, the PCA is used as receiver. The figure in dashed-line box illustrates the Schematic diagram of ultrafast THz s-SNOM. (B) The Raman scattering characterization of the Bi_2_Te_3_ sample, and the atomic structure of Bi_2_Te_3_ is shown in the insert of Figure 1B. (C) The XRD characterization of the Bi_2_Te_3_ sample.

The schematic diagram of the experimental setup is shown in Figure 1A. The ultrafast THz s-SNOM (NeaSpec, Germany) contains four modules: atomic force microscopy (AFM), static THz-TDS, OPTP, and TES. The entire system is driven by a fiber femtosecond laser (with a center wavelength of 1560 nm, a pulse width less than 100 fs, and a repetition frequency of 100 MHz) divided into three paths. Two laser beams are applied to the emitting photoconductive antenna and the detecting photoconductive antenna in the THz-TDS system. The last beam acts as the pump pulse, which is converted into a pulse with a central wavelength of 780 nm by a second-harmonic generation module. After passing through a beam expander and some mirrors, the pump pulse and the THz pulse are finally impinged onto the sample. During the experiment, the pump laser is incident obliquely onto the tip at an incident angle of 36°. The tip vibrates periodically above the sample for a frequency of 54 kHz with 40-nm diameter. When the pump pulse illuminating at the tip, an ultrahigh field enhancement will occur in the gap between the tip and the sample, and the sample absorbs the localized laser energy.^34^ More accurate near-field information can be obtained by extracting the high-order harmonics of the scattered field signal. All experiments were conducted at room temperature and in an atmospheric environment.

#### Sample preparation

In this study, high-quality Bi_2_Te_3_ thin films with varying thickness gradients were grown on 2-inch (0001) sapphire substrates using a molecular beam epitaxy (MBE) system with a base pressure of 10^−10^ Torr. The 0.43-μm double-side polished sapphire substrates were initially cleaned using a standard cleaning procedure. Following this, the substrates were annealed at 600°C for 40 min in the growth chamber to remove adsorbed gases. The growth of Bi_2_Te_3_ was performed using a two-step method. Initially, the substrate temperature was lowered to 160°C to grow a 1 quintuple layer (QL) to ensure adhesion of Bi_2_Te_3_ to the substrate. Subsequently, the substrate temperature was increased to 220°C for the growth of the remaining layers to ensure high quality of Bi_2_Te_3_. High-purity Bi (99.9999%) and Te (99.9999%) were evaporated from standard Knudsen cells, maintaining a flux ratio of 1:10 to ensure a Te-rich environment. The growth rate was maintained at 0.25 QL/min. The surface of the thin films was characterized *in situ* using reflection high-energy electron diffraction (RHEED) as shown in Figure S1, which displayed sharp streaks, indicating the high quality of the synthesized Bi_2_Te_3_.

The atomic structure schematic of Bi_2_Te_3_ on sapphire is shown in the insert of Figure 1B. Raman spectroscopy results, shown in Figure 1B, revealing two peaks at 96.86 cm^−1^ and 130.34 cm^−1^, corresponding to the $E_{g}^{2}$ and $A_{1g}^{2}$ modes of Bi_2_Te_3_, respectively, consistent with previously reported values.^35^ To obtain structural information about the samples, X-ray diffraction (XRD) with a wavelength of 0.15406 nm was used. The XRD pattern is presented in Figure 1C, where the highest intensity peak corresponds to the (0001) plane of the Al_2_O_3_ substrate. The remaining peaks from left to right correspond to the 003, 006, 0015, 0018, and 0021 planes of single-crystal Bi_2_Te_3_. All peak positions align with the standard positions,^36^ confirming the high-quality single-crystal structure of the grown Bi_2_Te_3_. The type of material under equilibrium conditions is n-type given from the transport measurement which is shown in supplemental information PART.2.

## Results and discussion

### THz-TDS and characterization of sample’s morphology

**Figure 2:**
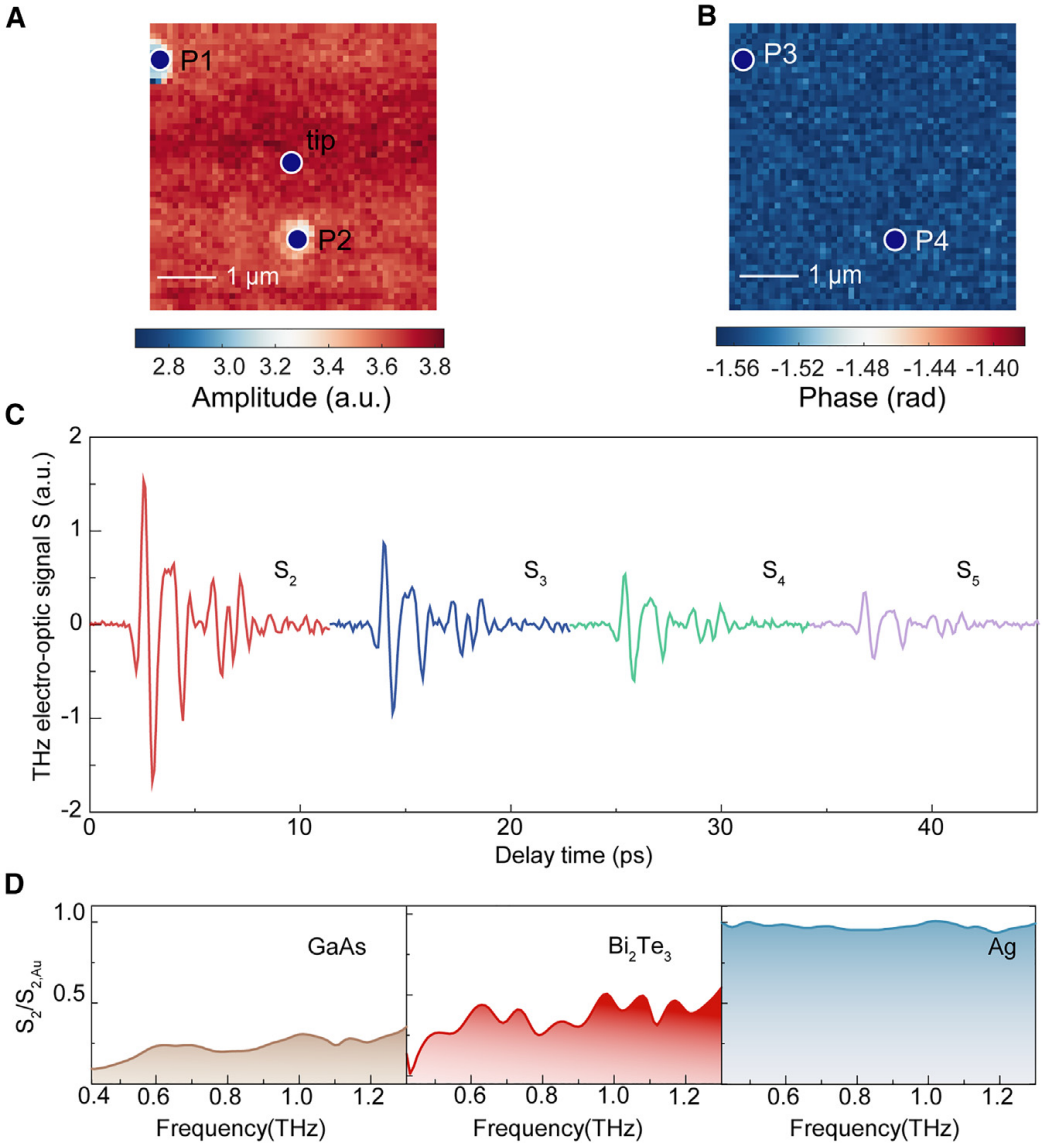
Static scattering scanning and topographic characterization of the sample (A) 5 μm × 5 μm amplitude scanning map of the second-order scattered signal. (B) 5 μm × 5 μm phase scanning map of the second-order scattered signal corresponding to Figure 2A. (C) The scattered signal at relatively flat area which is spotted at Figure 2A. (D) Normalized spectra of Bi_2_Te_3_, GaAs, and Ag referenced to the spectrum of Au. Scale bars, 1 μm.

In the initial step, we try to explore the equilibrium nanomorphology of Bi_2_Te_3_ film by THz-TDS. There exist two operation modes in THz-TDS process, a spectral mode and a scanning mode. The spectral mode acquires the complete time-domain scattering signal through a photoconductive antenna fixed at a specific location on the sample surface. The scanning mode is to track the n^th^ demodulated peak scattered signal while raster-scanning the tip across the sample. The static scanning THz-TDS can realize the function of sample characterization, similar to AFM, due to its sensitivity to the permittivity and morphology.^34^^,^^37^ Therefore, we utilized static scanning THz-TDS to determine and eliminate the influence of the change of morphology.^30^ Figure 2A shows the 5 μm × 5 μm amplitude scanning map of the second-order scattered signal and the phase scanning map of the corresponding signal as depicted in Figure 2B. Positions P1 and P2 represent two structural defects or dusts present on the surface of the amplitude scanning map. Correspondingly, P3 and P4 denote the corresponding positions in the phase scanning map. Significant differences between the defective regions and the flat areas are only observable in the amplitude scanning map, whereas the phase scanning map exhibits no significant distinction. This clearly suggests that the amplitude scanning map exhibits a higher sensitivity to the surface morphology compared to the phase scanning map. Based on the results of Figures 2A and 2B, the scattering scan image shows that the sample surface is relatively flat, with a few topographic defects or dusts in some areas. we selected the sample position at the center of the sample with the maximum scattering signal amplitude for data acquisition in the following experiments to avoid the effects from topographic defects as shown in Figure 2A. Figure 2C shows a typical THz scattered signal at a flat position of the sample surface.

To deeply explore the physical meaning of the scattered signal, we analyzed the scattering characteristics of Bi_2_Te_3_, with the metal Ag and semiconductor GaAs as references. We normalized the near-field response of Bi_2_Te_3_ with the standard sample Au for a more detailed analysis to eliminate the influence of near-field signal detection and artifacts from indirect far-field tip illumination.^38^ Figure 2D shows the normalized scattered signal amplitude diagrams of Bi_2_Te_3_, Ag, and GaAs. The spectral amplitude of the metal Ag shows its scattering property are similar to those of Au in THz region, while the semiconductor GaAs stays the lowest in the normalized spectrum amplitude with Bi_2_Te_3_ lying between the amplitudes of the former two.

By measuring the near-field THz scattered signal, the conductive characteristics of the samples in the THz regime can be compared intuitively. According to the finite dipole model (FDM),^39^^,^^40^ the normalized spectrum $\frac{S_{n}}{S_{n,Au}}$ is only related to the quasi-static reflection coefficient $\beta =\frac{\varepsilon -1}{\varepsilon +1}$ of the measured sample, the shape, material, and height of the tip, where n is the demodulation order and ε is the permittivity of the measured material. The sample exhibiting higher conductivity generally shows a larger reflection coefficient, which suggests that the normalized spectrum will approach the amplitude of Au and, in some instances, exceed it.^31^ This observation is corroborated by the normalized spectra of GaAs and Ag.

### Nanovideography of sample by OPTP and THz emission

**Figure 3:**
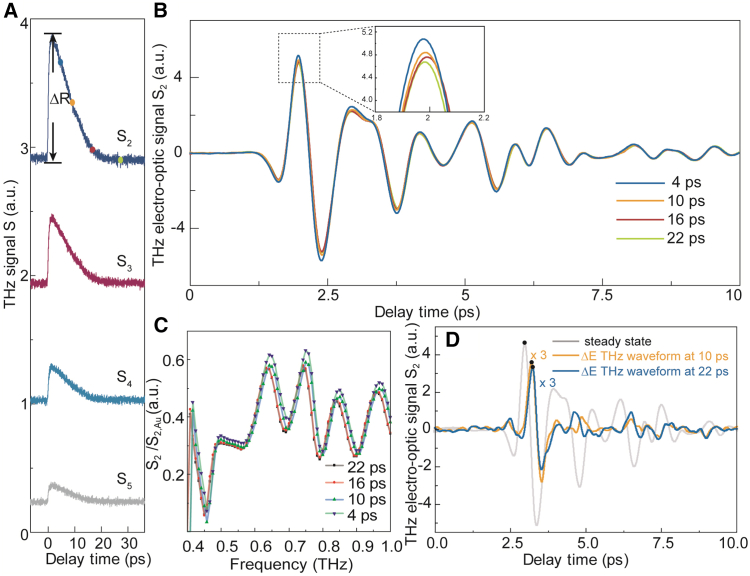
The OPTP results of Bi_2_Te_3_ with its pump-induced waveforms (A) The OPTP signals corresponding to demodulation orders 2–5. (B) The THz scattered waveforms corresponding to 4 ps, 10 ps, 16 ps, and 22 ps after 14.5 mw laser excitation. (C) The corresponding normalized spectra normalized by Au’s THz scattered waveform spectrum. (D) Steady-state(corresponding to −3 ps) and photo-induced terahertz near-field waveform(corresponding to 10 ps, 22 ps) at the second demodulation order.

To extend the ultrafast dynamics of Bi_2_Te_3_ at the nanoscale, we conducted near-field OPTP experiments at the position with the maximum static THz scattering amplitude, revealing the change in the transient conductivity of Bi_2_Te_3_ under photoexcitation. In this experiment, we sampled different THz scattered waveform at specific pump delay before and after photoexcitation to extract an OPTP curve which yields the spectral response as a function of the pump delay time. we obtained the OPTP signals corresponding to different demodulation orders at 14.5 mW pumping laser, as shown in Figure 3A. The signal amplitude decreases with the increase of the demodulation order, and the profile of the dropping part can be fitted perfectly with the single exponential decaying function $y=y_{0}+A\;\exp\left(-\left(t-t_{0}\right)/\tau \right)$ in all demodulation orders, which means there exist no other relaxation process. Each OPTP signals from different demodulation orders have the same 1/e relaxation time τ, which is nearly 10 ps. We chose several typical moments, 4 ps, 10 ps, 16 ps, and 22 ps after laser excitation, respectively to record the THz time-domain reflection signals, as illustrated in Figure 3B. Those signals were normalized as shown in Figure 3C, referenced to the spectrum of Au. Notably, in Figure 3B, there exists a minute time shift of pump-induced waveform between different delay. During the whole experiment, there existed no external influence being set up near the sample that will affect the time delay. To show this shift properly, we illustrated the photo-induced scattered terahertz waveform ΔE at 10 ps and 22 ps compared to the steady-state scattered waveform as shown in Figure 3D. The peak amplitude of the photo-induced waveform is significantly smaller than the total field attesting to the outstanding sensitivity. The slight time shift between 10 ps and 22 ps are nearly 37 fs which may link to the changes of dielectric function resulting from out-of-plane carrier of the sample.^38^

**Figure 4:**
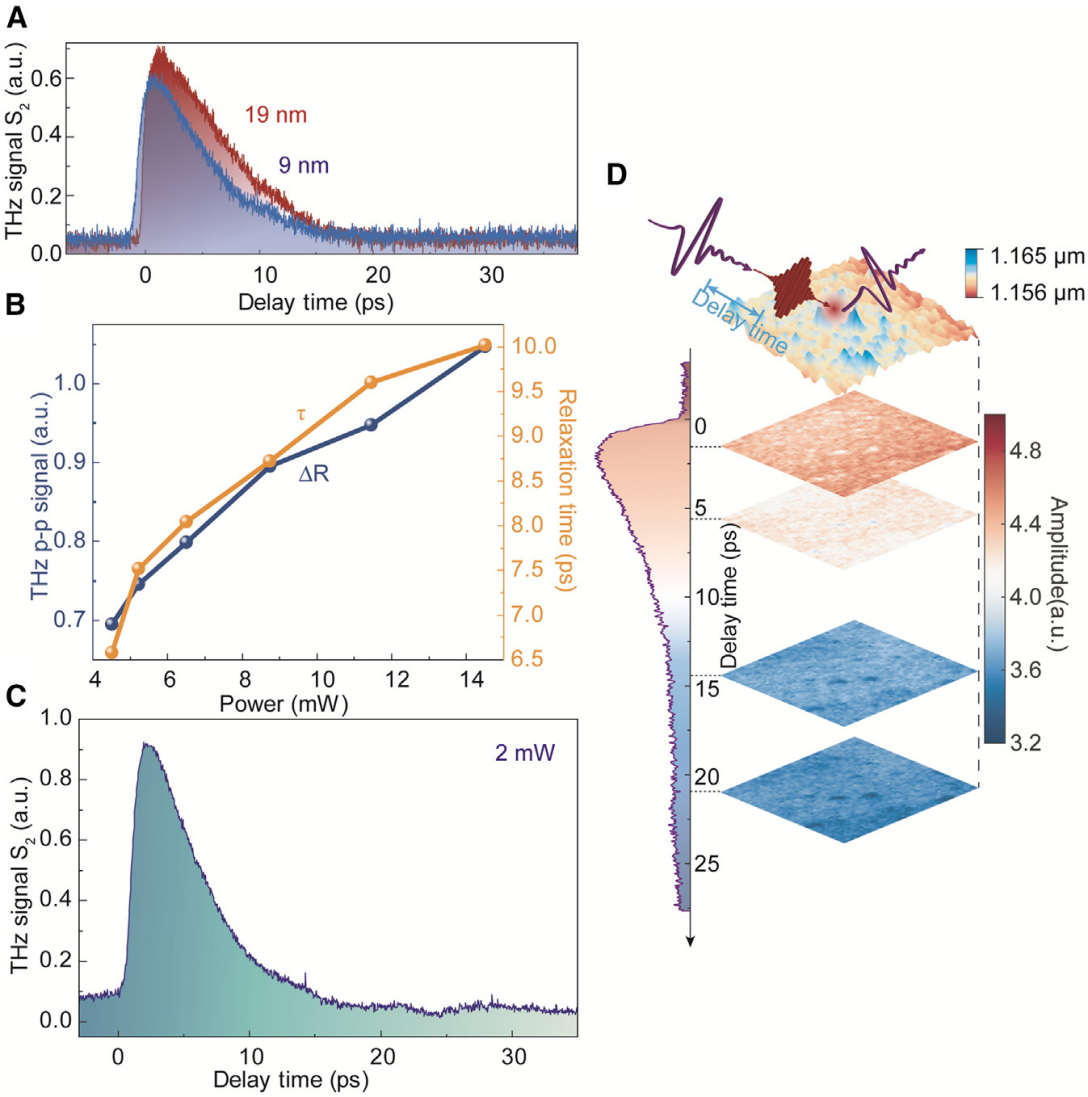
The OPTP signal dependences and THz scattering scanning images after laser pumping (A) The second demodulation order of OPTP signals at different thicknesses. (B) The peak-to-peak values of the THz reflection signals and the 1/e relaxation time of the 9 nm sample under different power excitations. (C) The OPTP signals under 2 mW laser pumping. (D) 5 μm × 5 μm THz scattering scanning images after laser pumping. The two-dimensional snapshots were obtained by fixing pump delay at different specific time.

The normalized spectra from the OPTP curve at each typical moment decrease with the increase of the pump delay, which reflects that the amplitude of the quasi-static reflection coefficient of Bi_2_Te_3_ is also decreasing. The THz reflectivity reaches the maximum value at approximately 600 fs during the laser pumping, which means the hot carriers thermalize within 600 fs through electron-electron scattering.^35^ After that, a nearly 10-ps relaxation process occurs which means that the electron-lattice interaction dominates the relaxation process giving from the scale of relaxation time.^35^^,^^41^ The OPTP signals of Bi_2_Te_3_ with different thicknesses were illuminated under a pump power of 14.5 mW, as shown in Figure 4A. As the thickness increases, under laser pumping, the maximum reflection of the THz signal by Bi_2_Te_3_ becomes larger, and the relaxation time also increases slightly. Figure 4B shows the measured OPTP signals of 9 nm Bi_2_Te_3_ when the power increases from 4.5 mW to 14.5 mW. In Figure 4B ΔR denotes the peak-peak amplitude of OPTP signals which represents the initial amplitude of electron-lattice interaction process. The increasing initial amplitude of the electron-lattice interaction process may be the result of the increasing number of carriers with growing power, showing the electron-lattice interaction gets greater and becomes more important with more pumping power. Observing OPTP signal with high signal-to-noise ratio (SNR) is possible even with a modest pump power of 2 mW as shown in Figure 4C, indicating that this method has the potential to be applied broadly and in a variety of situations.

As mentioned before, the THz scattering scanning images also play the role of sample characterization. Similarly, we obtained THz scattering scanning images at different times after the laser pulse pumping and observed the carrier relaxation time and spatial distribution at each point, as shown in Figure 4D. The corresponding scattering amplitude scanning images at 1 ps, 6 ps, 14 ps, and 22 ps after laser pumping respectively, indicating that the ultrafast THz scattering scanning image can reflect the morphological characteristics at any moment, and this scanning figure can exhibit its relaxation property in the spatial dimension. Therefore, it can be found that this kind of ultrafast THz scattering scanning image can become a powerful *in situ* characterization method, which can provide us with a fascinating perspective for observing the carrier dynamics of the sample. To demonstrate the detailed nanoscale resolution, we have obtained the OPTP curves around a large structural defect shown in Figure S3 which suggests that the resolution of ultrafast THz s-SNOM is at least 50 nm.

**Figure 5:**
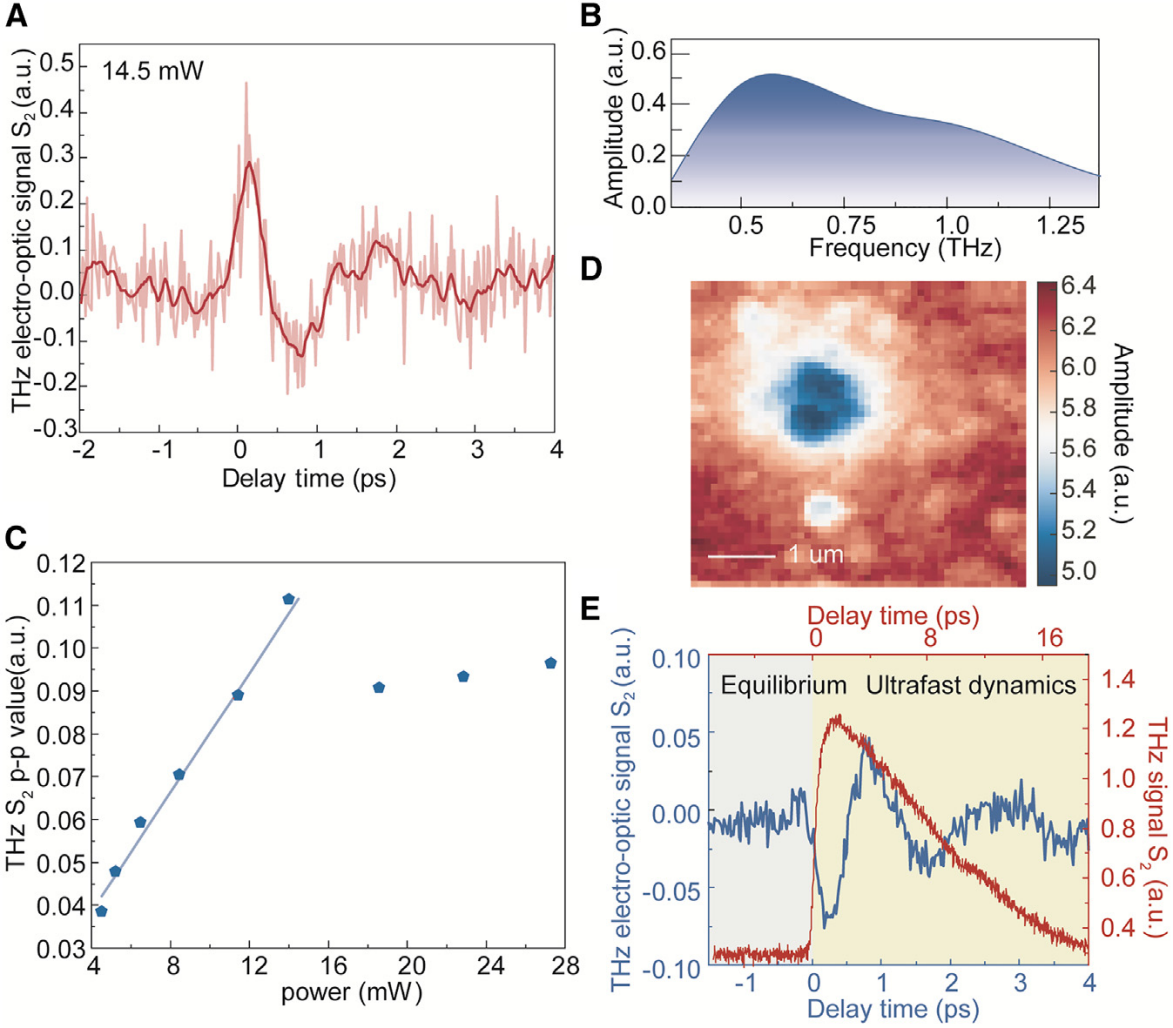
The THz emission of Bi_2_Te_3_ (A) The THz emission waveform of Bi_2_Te_3_ at 14.5 mW at second demodulation order. (B) The corresponding spectrum of the emission waveform. (C) The peak-to-peak terahertz emission signals at second demodulation order as a function of the laser power. (D) The THz scattering characterization of the sample after high-power laser illuminating. (E) The THz emission and the OPTP phenomenon occur at the same pumping time. Scale bars, 1 μm.

Under the same experimental conditions, we also observed the near-field THz emission signal of Bi_2_Te_3_ at the nanoscale. Figure 5A shows the second-order THz emission time-domain waveform corresponding to a pump power of 14.5 mW. It can be seen from the time-domain waveform in Figure 5A that the THz radiation produced by Bi_2_Te_3_ is a single-cycle pulse with a pulse width of ∼0.5 ps. Figure 5B displays the corresponding spectrum of the THz emission signal. We measured the power dependence of the THz emission signal as shown in Figure 5C and found that as the power increases, the second-order emission signal of Bi_2_Te_3_ shows a nearly linear rise. However, after illumination by higher power pump, the sample was destroyed after laser irradiation enhanced by the tip. Figure 5D shows the THz scattering scanning image after high-power laser irradiation^28^ which reflects the surface was damaged from its rough and uneven topography.

The pump laser excites Bi_2_Te_3_ at 0 ps. At this time, different phenomena were observed at the same delay through different experiments, as shown in Figure 5E. We combined the OPTP experiment with the THz emission experiment to analyze the ultrafast THz dynamics of TI at the nanoscale. Within 1 ps after laser excitation, we respectively observed that the rising stages of the THz emission pulse and the OPTP signal coincide, which enables us to observe the same process from different perspectives. The far-field equipment integrated with both OPTP and THz emission measurement can also achieve this function, but it cannot be used to obtain the information at the nanoscale. The ultrafast THz s-SNOM equipment provides more significant advantages for our more profound study of the ultrafast carrier dynamics and THz emission of nanomaterials.

### Conclusion

In summary, to gain more accurate information, we characterized the morphology of the TI Bi_2_Te_3_ by using a scanning near-field optical microscope coupled with an ultrafast THz-TDS system. Moreover, we observed and analyzed the OPTP with thickness- and power-dependence in the near field and nanoscale. The increasing final amplitude of the rising part of OPTP may be the result of the increasing number of carriers with growing power and thickness. The out-of-plane-carrier caused minute time shift in different pump delay attesting to the outstanding sensitivity in signal discrimination. We visually mapped the relaxation process and discovered that this method can be a promising *in situ* characterization technology to study the morphology and photocarrier dynamics with a resolution of nanometers. Even under small pumping power, we still gained good quality of OPTP signal. Besides, we observed THz emission from Bi_2_Te_3_ thin film and its power dependence. All these phenomena prove that Bi_2_Te_3_ can be used as a nanodevice, providing a reliable research basis for the miniaturization and integration of THz optoelectronic devices. In addition, we have verified that ultrafast THz s-SNOM is a reliable means to study the ultrafast dynamics of 2D materials due to its high sensitivity and high spatiotemporal resolution.

### Limitations of the study

In this study, due to the unique surface state of Bi_2_Te_3_, more precise FDM calculations were not performed to determine its permittivity. However, the extraction of permittivity may fall outside the scope of this work.

## Resource availability

### Lead contact

Further information and requests for resources and reagents should be directed to and will be fulfilled by the lead contact, Xiaojun Wu (xiaojunwu@buaa.edu.cn).

### Material availability

The study did not generate any unique reagents.

### Data and code availability

• All data reported in this paper will be shared by the lead contact upon request.
• This paper does not report original code.
• Any additional information required to reanalyze the data reported in this paper is available from the lead contact upon request.

## Acknowledgments

This work is supported by the 10.13039/501100001809National Natural Science Foundation of China (U23A6002, 92250307), 10.13039/501100012166National Key R&D Program of China (2022YFA1604402), the Open Project Program of Wuhan National Laboratory for Optoelectronics NO.2022WNLOKF006.

## Author contributions

X.J.W. conceived the idea of nanoscale ultrafast dynamic of TI, Z.Y.H. carried out the experiments, collected and analyzed the data. J.L. completed the growth of samples and their characterizations. P.Y.L., D.L., M.C.D., and Z.J.R. completed the drawing work. J.H.C. contributed with helpful discussion. Z.Y.H. wrote the manuscript with revisions by all.

## Declaration of interests

The authors declare no conflict of interest.

## STAR★Methods

### Key resources table

**Table:** 

REAGENT or RESOURCE	SOURCE	IDENTIFIER
**Chemicals, peptides, and recombinant proteins**		

Tellurium ingot,99.9999%	Prmat metal,materials&analysis	Cat#CAS: 13494-80-9
Bismuth ingot,99.9999%	Prmat metal,materials&analysis	Cat# CAS: 7440-69-9
Germanium ingot,99.9999%	Prmat metal,materials&analysis	Cat# CAS: 7440-56-4

**Software and algorithms**		

origin	Originlab	www.originlab.com/
Gwyddion	Department of Nanometrology, Czech Metrology Institute	http://gwyddion.net/
Python	Python Software Foundation	https://www.python.org

**Other**		

X-ray diffraction system	bruker	https://www.bruker.com/
molecular beam epitaxy system	ssi-products	http://www.ssi-physics.com/
reflection high-energy electron diffraction system	staib instruments	https://www.staibinstruments.com/
Ultrafast terahertz scattering-type scanning near-field optical microscope	neaspec	https://www.attocube.com/en/products/microscopes/nanoscale-imaging-spectroscopy/THz-neaSCOPE-s#

### Experimental model and study participant details

The size of our samples are approximately 1.5 cm × 1.5 cm. In the stage of extracting the equilibrium state, we use the scanning mode to raster-scan the sample for obtaining its morphology and scattering characteristics. The scanning process costs 8 minutes per picture. The integration time is set to 100 ms, the tapping amplitude to 226 nm, and the radius of the tip is approximately 20 nm. For single-point TDS signal extraction, the integration time is set to 200 ms and the averaging time is set to 10.

In the stage of obtaining the OPTP signals and the THz emission signals, we use the same scanning parameters as in the first stage. For single-point OPTP signal extraction, the integration time is set to 50 ms and the averaging time is set to 1. For single-point THz emission signal extraction, the integration time is set to 200 ms and the averaging time is set to 1. According to FDM, the scattered signals are normalized by dividing the scattered THz signal of gold.

### Method details

#### Finite dipole model

The interaction between incident light, tip and material can be described by finite dipole model.

The scattering coefficient S can be described as the following equation:

(Equation 1) $$S=\frac{E_{s}}{E_{i}}={\left(1+r\right)}^{2}\alpha_{eff}$$

In Equation 1, $E_{s},E_{i}$ represents scattered wave and incident wave. Equation 1 can be divided into two parts. The first part is far-field factor ${\left(1+r\right)}^{2}$. r is Fresnel reflection coefficient. The far-field factor can be regarded as constant in most of the cases. Another part is effective polarizability $\alpha_{eff}$ which stands for tip-sample interaction. It can be described as:

(Equation 2) $$\alpha_{eff}\propto 1+\frac{f_{0}}{2\left(1-f_{1}\beta \right)}$$

(Equation 3) $$f_{0,1}=\left(g-\frac{r_{t}+2H+W_{0,1}}{2L}\right)\frac{\ln\left(4L\right)-\ln\left(r_{t}+4H+2W_{0,1}\right)}{\ln\left(4L\right)-\ln\;r_{t}}$$

g=0.7exp(0.06i), H is the height between the tip and sample which is vibrating with frequency of 54 kHz. $r_{t}$ is the radius of the tip. $W_{0}\approx 1.31r_{t},W_{1}\approx 0.5r_{t}$. L is the effective length of tip. $f_{0,1}$ are geometric factors which are independent with sample. By obtaining effective polarizability, we can conduct the measured sample’s quasi-static reflection coefficient $\beta =\frac{\varepsilon -1}{\varepsilon +1}$ to get its permittivity.

During our testing, to get more precise signal, the tip oscillates periodically. Then we use a lock-in amplifier to demodulate signal at higher harmonics of that frequency, the demodulation signal can be written as $S_{n}={\left(1+r\right)}^{2}\alpha_{eff,n}$.

In order to avoid the influence from detector and other unknown factors. We usually normalize the demodulation signal with a reference sample like Au or Si. This procedure can be described as:

(Equation 4) $$\frac{S_{n}}{S_{n,ref}}=\frac{E_{s,n}}{E_{s,n,ref}}=\frac{{\left(1+r\right)}^{2}\alpha_{eff,n}}{{\left(1+r_{ref}\right)}^{2}\alpha_{eff,n,ref}}$$

If the far-field terms are nearly the same, it can be cancelled as well:

(Equation 5) $$\frac{S_{n}}{S_{n,ref}}\approx \frac{\alpha_{eff,n}}{\alpha_{eff,n,ref}}$$

Moreover, as for reference sample Au, $\beta_{Au}\approx 1$. This will make it easier to analyze the signal.

### Quantification and statistical analysis

To make quantification, we employed Python, Origin and, Gwyddion, and the pre-installed software of SNOM to analyze our data.
