# Supplementary material for: Nanoscale ultrafast dynamics in Bi2Te3 thin film by terahertz scanning near-field nanoscopy
Source: iScience. 2025 Jan 20;28(2):111840. doi: 10.1016/j.isci.2025.111840 (PMC11841264; doi:10.1016/j.isci.2025.111840)
Supplement: Document S1. Figures S1–S3 [file mmc1.pdf]

iScience, Volume *28*

Supplemental information

Nanoscale ultrafast dynamics in Bi_2Te_3 thin film by terahertz scanning near-field nanoscopy

Ziyu Huang, Jing Li, Peiyan Li, Lin Du, Mingcong Dai, Jiahua Cai, Zejun Ren, Tianxiao Nie, and Xiaojun Wu

Supplemental Information

PART. 1 the image of reflection high-energy electron diffraction (RHEED)

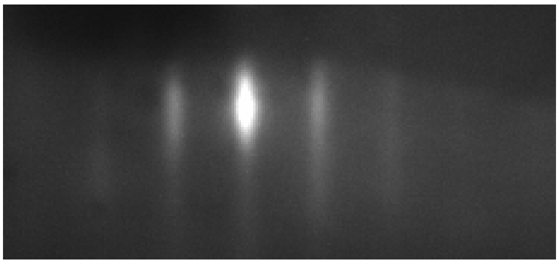

Figure S1 the RHEED image

PART. 2 transport measurement

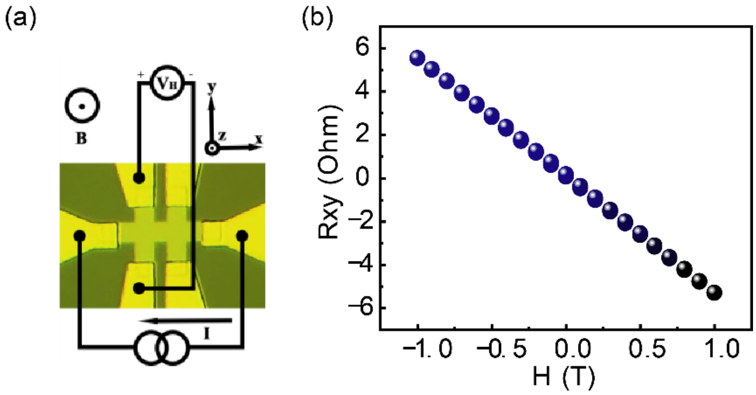

Figure S2 transport properties of Bi_2Te_3 (a) Optical picture of transport measurement (b) Hall resistivity curve

PART. 3 nanoscale resolution of OPTP signals

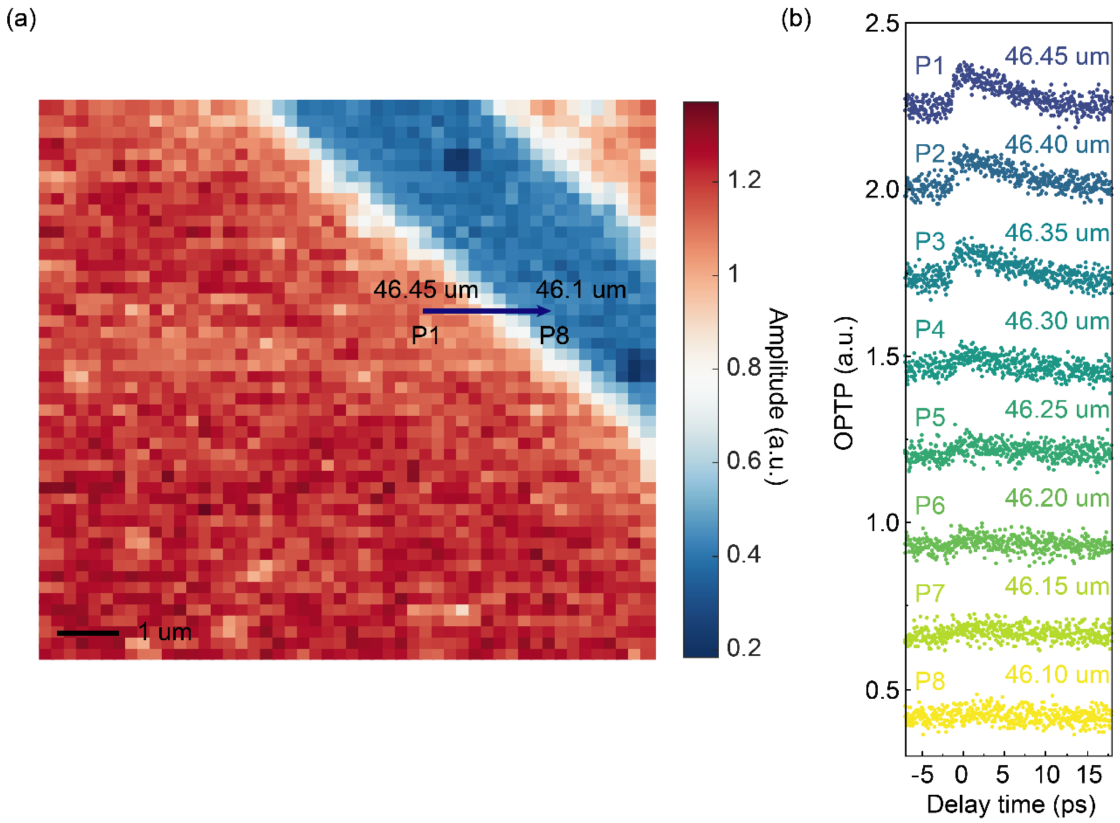

Figure S3 The OPTP curve in different positions around a large structural defect (a) The amplitude scanning image (b) The 2nd demodulation order of OPTP curves per points shown in (a), the distance between each point is 50 nm.
